# Differences in activated clotting time and total unfractionated heparin dose during pulmonary vein isolation in patients on different anticoagulation therapy

**DOI:** 10.1002/clc.23681

**Published:** 2021-07-01

**Authors:** Ivan Zeljkovic, Sandro Brusich, Daniel Scherr, Vedran Velagic, Vassil Traykov, Andrej Pernat, Ante Anic, Janko Szavits Nossan, Matevz Jan, Zoran Bakotic, Borka Pezo Nikolic, Vjekoslav Radeljic, Ana Bojko, Ivica Benko, Sime Manola, Nikola Pavlovic

**Affiliations:** ^1^ Department of Cardiology Sestre milosrdnice University Hospital Centre Zagreb Croatia; ^2^ Department of Cardiology University Hospital Centre Rijeka Rijeka Croatia; ^3^ Department of Cardiology Medical University Graz Graz Austria; ^4^ Department of Cardiology University Hospital Centre Zagreb Zagreb Croatia; ^5^ Department of Cardiology Acibadem City Clinic Tokuda Hospital Sofia Bulgaria; ^6^ Department of Cardiology University Hospital Centre Ljubljana Ljubljana Slovenia; ^7^ Department of Cardiology University Hospital Centre Split Split Croatia; ^8^ Department of Cardiology Magdalena Clinic KrapinskeToplice Croatia; ^9^ Department of Cardiac Surgery University Hospital Centre Ljubljana Ljubljana Slovenia; ^10^ Department of Cardiology General Hospital Zadar Zadar Croatia; ^11^ Department of Cardiology University Hospital Dubrava Zagreb Croatia

**Keywords:** apixaban, atrial fibrillation, dabigatran, pulmonary vein isolation, rivaroxaban, warfarin

## Abstract

**Background:**

Periprocedural pulmonary vein isolation (PVI) anticoagulation requires balancing between bleeding and thromboembolic risk. Intraprocedural anticoagulation is monitored by activated clotting time (ACT) with target value >300 s, and there are no guidelines specifying an initial unfractionated heparin (UFH) dose.

**Methods:**

We aimed to assess differences in ACT values and UFH dosage during PVI in patients on different oral anticoagulants. We conducted an international, multi‐center, registry‐based study. Consecutive patients with atrial fibrillation (AF) undergoing PVI, on uninterrupted anticoagulation therapy, were analyzed. Before transseptal puncture, UFH bolus of 100 IU/kg was administered regardless of the anticoagulation drug.

**Results:**

Total of 873 patients were included (median age 61 years, IQR 53–66; female 30%). There were 248, 248, 189, 188 patients on warfarin, dabigatran, rivaroxaban, and apixaban, respectively. Mean initial ACT was 257 ± 50 s, mean overall ACT 295 ± 45 s and total UFH dose 158 ± 60 IU/kg. Patients who were receiving warfarin and dabigatran compared to patients receiving rivaroxaban and apixaban had: (i) significantly higher initial ACT values (262 ± 57 and 270 ± 48 vs. 248 ± 42 and 241 ± 44 s, *p* < .001), (ii) significantly higher ACT throughout PVI (309 ± 46 and 306 ± 44 vs. 282 ± 37 and 272 ± 42 s, *p* < .001), and (iii) needed lower UFH dose during PVI (140 ± 39 and 157 ± 71 vs. 171 ± 52 and 172 ± 70 IU/kg).

**Conclusion:**

There are significant differences in ACT values and UFH dose during PVI in patients receiving different anticoagulants. Patients on warfarin and dabigatran had higher initial and overall ACT values and needed lower UFH dose to achieve adequate anticoagulation during PVI than patients on rivaroxaban and apixaban.

## 1 INTRODUCTION

Pulmonary vein isolation (PVI) is a well‐established therapeutic option for patients with atrial fibrillation (AF).^1^, ^2^ Although currently performed on a routine basis, PVI is associated with a nonnegligible complication rates.^3^, ^4^ Periprocedural PVI anticoagulation strategy always represents a balance between the risk of bleeding (vascular access site complications and pericardial effusion/tamponade) and the risk of thromboembolic incidents, in particular cerebrovascular events which incidence could reach up to 1%.^1^, ^2^, ^3^, ^4^, ^5^, ^6^ Effective intraprocedural anticoagulation is essential to minimize the risk of thromboembolism during the PVI and is monitored throughout the procedure by activated clotting time (ACT).^1^, ^7^, ^8^, ^9^ It has been observed that thrombi could form on the transseptal sheath and/or the catheter even before the transseptal puncture.^7^ Early unfractionated heparin (UFH) administration significantly reduces the risk for thrombus formation.^7^, ^8^ However, the use of high UFH loading doses may come with a higher bleeding risk, suggesting that there exists a potential for overshooting with the initial UFH bolus.^1^, ^4^, ^10^, ^11^ The EHRA/HRS consensus statement recognizes the need of higher initial doses of UFH in patients on DOACs than on vitamin K antagonist (VKA), as well as the requirement for more frequent ACT measurements.^1^ Similarly, it has been recognized by the EHRA Practical guide on the use of DOACs, that larger doses of UFH might be required to achieve target ACT values in patients on DOACs than on VKA.^12^ In addition, recent studies showed great variability in UFH loading dosage and periprocedural anticoagulation strategies.^1^, ^4^, ^8^, ^9^, ^10^, ^11^, ^12^ Thus, the aim of the current study is to assess differences in ACT values and total UFH dosage during PVI in patients on different oral anticoagulation therapies. We aimed to evaluate the differences in the initial and overall ACT during the procedure as well as doses of the initial UFH bolus required to achieve ACT >300 s in patients receiving different oral anticoagulation therapy.

## 2 METHODS

We performed an international, multi‐center, registry‐based cohort analysis. Total of nine electrophysiology centers from four countries (Table 1) were actively participating in the prospective Southeast‐Central European PVI (SECE‐PVI) registry. Consecutive patients with paroxysmal, persistent and long‐standing persistent AF enrolled in the SECE‐PVI registry, in the period between April 2016 and July 2019, were analyzed. Patients with moderate and severely decreased renal function (creatinine clearance rate < 50 ml/min), those with anticoagulation therapy started just before or after PVI and in whom the ACT measurements were not done or not recorded properly were excluded from the study. Additionally, patients with left atrial appendage thrombus have not undergone PVI, and thus could not be included in the registry or the study itself. Baseline demographic characteristics, medical history with chronic medication usage and all procedural data were collected. Baseline laboratory data included hemoglobin, platelet count, international normalized ratio (INR) and serum creatinine.

**TABLE 1:** Electrophysiology centers participating in the study with number of patients per center

Centre	*N* of patients
University Hospital Sestre Milosrdnice (Zagreb, Croatia)	388
General Hospital Zadar (Zadar, Croatia)	19
University Hospital Zagreb (Zagreb, Croatia)	91
University Hospital Rijeka (Rijeka, Croatia)	104
Clinic for Cardiovascular Medicine Magdalena (KrapinskeToplice, Croatia)	54
University Medical Center Ljubljana, Cardiology (Ljubljana, Slovenia)	6
University Medical Center Ljubljana, Cardiac Surgery (Ljubljana, Slovenia)	51
Tokuda Hospital Sofia (Sofia, Bulgaria)	63
University Hospital Graz (Graz, Austria)	97

All included patients gave written informed consent for participating in the SECE‐PVI registry. The hospital's Ethics Committee gave its approval for the study, which was conducted in accordance with the current version of Declaration of Helsinki.

### 2.1 PVI procedure

Transthoracic and transoesophageal echocardiogram to rule out left atrial (LA) thrombus, to determine LA diameter in the parasternal long axis (PLAX) plane, and left ventricular ejection fraction (LVEF) were performed before every PVI procedure. PVI procedure was performedeither using: (i) focal irrigated‐tip radiofrequency (RF) catheter (RF‐group) in combination with a 3D electroanatomical mapping systems (either CARTO3, Biosense Webster, or NavX, Abbott) as described in detail previously^13^, ^14^; (ii) using the 2nd‐generation cryoballoon (Arctic Front Advance 28 mm Medtronic Inc.,) ablation (CB‐group) as described in detail previously.^13^, ^14^

### 2.2 Anticoagulation therapy and ACT measurement

In each group, last dose of warfarin or DOAC was given in the evening before the procedure. When PVI was performed in the afternoon, morning dose of warfarin or DOAC was administered in the morning on the day of the procedure. Before transseptal puncture, initial UFH bolus ‐ 100 U/kg was administered intravenously regardless of the anticoagulation drug.^11^ After UFH bolus administration, to maintain the ACT >300 s throughout the entire procedure, subsequent dosing of UFH was dictated by physician's preference. ACT was measured in 15‐minute intervals as a point‐of‐care‐test using ACT Plus® (Medtronic Inc., Minneapolis, MN, USA). The ACT values after the initial UFH in units and units/kg, the total units and units/kg of UFH given to achieve an ACT >300 s, and number of UFH boluses given to achieve ACT >300 s were recorded. Overall ACT was defined as a mean value of all ACT values measured in one patient during the PVI procedure. Initial ACT was defined as a first ACT value done 15 min after the initial UFH bolus dose, and maximal (max) ACT value as a maximal value measured during the procedure for one patient.

### 2.3 Statistical analysis

The distribution of variables was tested using Kolmogorov–Smirnov test. Continuous variables are presented as mean ± *SD* when variable's distribution was normal and as median with interquartile range (IQR) when variable's distribution was skewed. For continuous variables, comparisons were made using Student's *t* test as parametric test for independent samples, or using Mann–Whitney *U* test as nonparametric test for independent samples. Categorical variables are presented as absolute numbers and/or percentages. Discrete variables were compared using Fisher's exact test.

A *p* value of <.05 was prespecified to indicate statistical significance. Statistical analysis was performed using SPSS version 20 (IBM SPSS Statistics, Armonk).

## 3 RESULTS

A total of 873 AF patients, on uninterrupted anticoagulation therapy, in whom ACT measurement during PVI was done, were included (median age 61 years; IQR 53–66; female 30%; BMI 28.5 ± 4.1 kg/m^2^, LVEF 60%, PLAX 42 IQR 39‐46 mm). There were 248 (28.4%), 248 (28.4%), 189 (21.7%), 188 (21.5%) patients on warfarin, dabigatran, rivaroxaban, and apixaban, respectively. Mean initial ACT (15 min after UFH bolus) was 257 ± 50 s, mean overall ACT 295 ± 45 s and the mean total UFH dose per kg 158 ± 60 IU/kg. There were no differences among patients receiving different anticoagulation therapy (warfarin, dabigatran, rivaroxaban, apixaban) regarding demographic data, body mass index, total procedure, and left atrial dwell time (Table 2).

**TABLE 2:** Demographic and procedural data of patients included in the study

	Total (*n* = 873)	Warfarin (*n* = 248)	Dabigatran (*n* = 248)	Rivaroxaban (*n* = 189)	Apixaban (*n* = 188)	*p* value
Demographics						
Age (years)	61 (53–66)	60 (51–66)	59 (52–65)	58 (50–66)	58 (49–67)	0.81
Female (%)	31 (269)	31 (78)	25 (63)	30 (56)	38 (72)	0.13
BMI (kg/m^2^)	28.5 ± 4	28.9 ± 4.2	28.3 ± 3.9	28.3 ± 3.6	28.5 ± 4.2	0.70
Weight (kg)	89 ± 15	89 ± 14	90 ± 15	89 ± 15	88 ± 15	0.68
History (%)						
Hypertension	65 (563)	73 (180)	60 (149)	62 (118)	62 (116)	.06
Diabetes mellitus	8.9 (78)	11 (28)	8.9 (22)	10 (19)	4.8 (9)	0.11
Hyperlipidemia	47 (412)	48 (119)	52 (129)	40 (75)	47 (89)	.08
Smoking	24 (209)	28 (69)	24 (60)	20 (37)	23 (43)	0.51
Stroke/TIA	5.5 (48)	4.0 (10)	6.9 (17)	5.8 (11)	4.8 (9)	0.45
CAD	8.8 (77)	8.1 (20)	8.5 (21)	11 (21)	7.4 (14)	0.65
MI	4.1 (36)	5.2 (13)	3.6 (9)	3.7 (7)	3.7 (7)	0.65
COPD	1.9 (17)	2.4 (6)	1.6 (4)	1.1 (2)	2.7 (5)	0.63
OSAS	1.7 (15)	0.8 (2)	0.8 (2)	2.6 (5)	2.7 (5)	0.22
Renal failure (CrCl>50 and < 90 ml/min)	3.3 (29)	4.4 (11)	2.4 (6)	2.1 (4)	4.3 (8)	0.39
HAS‐BLED score	0.86 ± 0.86	0.99 ± 0.89	0.82 ± 0.86	0.77 ± 0.82	0.83 ± 0.83	0.63
CHA_2_DS_2_‐VASc score	1.68 ± 1.21	1.75 ± 1.18	1.68 ± 1.19	1.60 ± 1.22	1.68 ± 1.28	0.30
Echocardiography						
LA diameter (mm)	42 (39–46)	42 (37–47)	44 (39–47)	42 (38–45)	42 (38–46)	.07
LVEF (%)	60 (55–65)	60 (55–65)	60 (55–65)	60 (50–65)	60 (55–65)	0.37
Laboratory results						
Hemoglobin (g/L)	145 (136–153)	144 (137–151)	146 (133–155)	144 (136–154)	146 (137–152)	0.28
Platelet count (*10^9^/L)	214 (181–247)	214 (178–251)	218 (186–255)	204 (181–248)	216 (180–247)	0.13
INR	1.51 ± 1.62	2.17 ± 0.55	1.14 ± 0.24	1.37 ± 0.33	1.08 ± 0.48	<.001
Creatinine (μmol/L)	87 (75–98)	90 (70–110)	86 (75–97)	85 (72–101)	84 (71–104)	.06
Procedural data						
Ablation modality						.06
RF ablation	71 (621)	76 (189)	66 (164)	75 (141)	68 (127)	
CB ablation	29 (252)	24 (59)	34 (84)	25 (48)	32 (61)	
Total procedure time (min)	119 ± 47	118 ± 48	125 ± 51	114 ± 45	120 ± 49	0.13
Left atrial dwell time (min)	83 ± 33	80 ± 33	88 ± 35	84 ± 34	86 ± 33	0.15

Abbreviations: BMI, body mass index; CAD, coronary artery disease; CB, cryoballoon; COPD, chronic obstructive pulmonary disease; INR, international normalized ratio; LA, left atrium; LVEF, left ventricle ejection fraction; MI, myocardial infarction; OSAS, obstructive sleep apnea syndrome; RF, radiofrequency; TIA, transitory ischemic attack.

Patients who were on warfarin had mean initial, maximal and overall ACT values of 262 ± 57 s, 340 ± 54 s, and 309 ± 46 s. Patients who were on dabigatran had mean initial, maximal and overall ACT values of 270 ± 48 s, 330 ± 51 s, and 306 ± 43 s. Patients who were on rivaroxaban had mean initial, maximal and overall ACT values of 248 ± 42 s, 305 ± 45 s, and 282 ± 37 s. Patients who were on apixaban had the lowest mean initial, maximal and overall ACT values (241 ± 44 s, 293 ± 48 s, and 272 ± 41 s), respectively. Patients who were on warfarin had significantly higher initial ACT values in comparison to patients on rivaroxaban and apixaban (262 ± 57 vs. 248 ± 42 and 241 ± 44 s, *p* < .001), but not in comparison to patients on dabigatran (262 ± 57 vs. 270 ± 48 s, *p* = 0.45) (Table 3). Also, patients on warfarin had a higher overall ACT throughout the PVI (309 ± 46 vs. 282 ± 37 and 272 ± 42 s, *p* < .001) in comparison to patients on rivaroxaban and apixaban, but not in comparison to patients on dabigatran (309 ± 46 vs. 306 ± 44 s, *p* = 0.54) (Table 3). Moreover, target ACT value after the initial (bolus) UFH dose (measured 15 min after) was reached in 20% (51/249), 25% (62/248), 13% (24/189), 10% (19/188) of patients who were on warfarin, dabigatran, rivaroxaban, and apixaban, respectively (Table 3). In addition, target ACT value was reached within the end of the procedure in significantly higher share of patients on warfarin and dabigatran in comparison to patients on rivaroxaban and apixaban (81% vs. 74% vs. 60% vs. 49%, *p* < .001) (Table 3).

**TABLE 3:** Unfractionated heparin doses and intraprocedural ACT values in patients on warfarin and different direct oral anticoagulant drugs

	Warfarin (*n* = 248)	Dabigatran (*n* = 248)	Rivaroxaban (*n* = 189)	Apixaban (*n* = 188)	*p* value
Intraprocedural anticoagulation					
Initial UFH dose (IU)	8894 ± 1442	8982 ± 1497	8888 ± 1542	8853 ± 1491	0.68
Total UFH (IU/kg)	140 ± 39	157 ± 71	171 ± 52	172 ± 70	**<.001**
Initial ACT (sec)	262 ± 57	270 ± 48	248 ± 42	241 ± 44	**.02**
Maximal ACT (sec)	340 ± 54	330 ± 51	305 ± 45	293 ± 48	**<.001**
Overall ACT (sec)	309 ± 46	306 ± 43	282 ± 37	272 ± 41	**<.001**
Target ACT reached after initial UFH dose (% patient)	20 (51/249)	25 (62/248)	13 (24/189)	10 (19/188)	**<.001**
Target ACT reached during PVI (% patients)	81 (201/249)	74 (184/248)	60 (113/189)	49 (92/188)	**<.001**

Abbreviations: ACT, activated clotting time; IU, international units; kg, kilogram; sec, seconds; UFH, unfractionated heparin.

They are all <0.001, except initial ACT is 0.02.

Initial UFH doses did not differ between the study groups (*p* = 0.68), however, patients on warfarin needed significantly lower total UFH dose during PVI in comparison to patients on rivaroxaban and apixaban (140 ± 39 vs. 171 ± 52 and 172 ± 70 IU/kg, *p* < .001), and similar dose to patients on dabigatran (140 ± 39 vs. 157 ± 71 IU/kg, *p* = 0.19) (Table 3).

## 4 DISCUSSION

The main findings of this multi‐center, registry‐based cohort study are as follows: (1) there are significant differences in achieved ACT values during the PVI procedure depending on the anticoagulation drug used. Patients who were on warfarin and dabigatran in comparison to patients on rivaroxaban and apixaban had: (i) significantly higher initial ACT values (262 ± 57 and 270 ± 48 vs. 248 ± 42 and 241 ± 44 s, *p* <0.001), (ii) significantly higher mean ACT throughout the PVI (309 ± 46 and 306 ± 44 vs. 282 ± 37 and 272 ± 42 s, *p* < .001); (iii) significantly lower doses of UFH were required in patients receiving warfarin and dabigatran to aim recommended ACT values (140 ± 39 and 157 ± 71 vs. 171 ± 52 and 172 ± 70 IU/kg, *p* < .001).

The requirement for higher initial doses of UFH during PVI in patients receiving DOACs has been previously reported.^11^, ^15^, ^16^, ^17^, ^18^ Recently, the study by Payne et al.^10^ showed that the patients receiving DOACs require significantly higher doses of heparin to achieve ACT >300 s, with no difference in UFH dose and achieved ACT between different DOACs. However, in their study, only 11 patients of 89 taking DOACs were on dabigatran.

Also, current consensus documents and guidelines acknowledge that there is a difference in UFH doses required to achieve recommended ACT values in patients using DOACs and VKA.^1^, ^12^, ^13^ Different doses of initial UFH bolus could be used for patients on VKA and DOACs with up to 20% higher doses recommended for all DOACs.^1^ In our study, the centers administered 100 units of UFH per kg, similar to majority of centers as reported in the ESC‐EHRA AF ablation registry.^3^

However, our study has shown that there was a difference in mean and maximum ACT achieved between different DOACs and warfarin and that higher doses of UFH was necessary to achieve recommended values of ACT during PVI. In patients treated with dabigatran, ACT values were significantly higher than in patients treated with apixaban or rivaroxaban, and required significantly lower doses of UFH to achieve the target ACT.

The results of our study corroborate the results of randomized trials comparing different DOACs vs warfarin in patients undergoing PVI.^15^, ^16^, ^17^, ^18^ The heparin doses required to achieve target ACT values and mean ACT values were similar for dabigatran and warfarin in RE‐CIRCUIT,^15^ while higher doses of heparin were required to achieve target ACT and mean ACT values were lower in patients on apixaban, edoxaban and rivaroxaban than on VKA in AXAFA AFNET 5,^16^ ELIMINATE AF^17^ and VENTURE AF^18^ trials.

Current recommendations are to maintain ACT >300 s during the PVI procedure^1^, ^13^ to prevent thromboembolic complications. And while it is clear that achieving and maintaining ACT >300 s is paramount to decrease risk of stroke during PVI,^19^ it should be recognized that the use of higher heparin loading doses may come with a risk. Notably, in a study by Dessault et al.,^10^ among 145 patients undergoing left sided procedures, patients with a hemorrhagic complication, had a mean initial ACT 397.33 s suggesting that there exists a potential for overshooting with a higher initial heparin bolus which is not inconsequential. Similarly, ELIMINATE AF trial^17^ speculated that the higher doses of UFH in edoxaban group might have contributed to higher incidence of bleeding complications found in this study.

ACT as a global coagulation test is sensitive to abnormalities in the intrinsic and common coagulation pathway. Therefore, it would be expected that both VKA and different NOACs have effects on ACT. From previous reports in differences in ACT levels between different DOACs, the higher ACT values with dabigatran might be explained by dose dependent effect dabigatran has on ACT, whereas therapeutic doses of factor Xa inhibitors do not show such effect.^13^ Also, recent ACTARD ex vivo study, found that UFH has similar effects on ACT values in patients taking dabigatran and VKA, while ACT strongly underestimates effects of UFH in patients on factor Xa inhibitors, especially apixaban, leading to potential UFH dose overshooting in these patients.^20^ Additionally, an in vitro study by Dincq et al. has shown that dabigatran interferes more with ACT than do FXa inhibitors.^21^ Therefore, there is unresolved question whether ACT measurement represents adequate monitoring tool of UFH effect during PVI procedure in patients on DOACs (especially FXa inhibitors). Further studies are required to define target ACT values or different monitoring strategies in this group of patients. Based on our results and adhering to current recommendations regarding target ACT values, it might be reasonable to use higher initial doses of UFH in patients treated with apixaban or rivaroxaban than in patients on VKA or dabigatran.

### 4.1 Limitations

There are several limitations to the current study. First, dosing of UFH after each ACT measurement was at operator's discretion so that differences in additional UFH dosing might have had an impact on the results. Second, patients with renal insufficiency (CrCl<50 ml/min) were excluded from the study, so the results and conclusions should not be applied to this group of patients. Also, there are no data on NOAC doses in the data set which might have affected the results. However, since patients with renal insufficiency were excluded as well as that the patients were treated according to current guidelines, we can assume, that most of the patients have been taking therapeutic doses of DOACs. Finally, patients taking edoxaban were not included since edoxaban at the time was not registered in the countries of participating centers.

## 5 CONCLUSION

There are significant differences in ACT values and UFH requirements for achieving targeted ACT during PVI procedures in patients receiving different anticoagulation therapy. Patients on warfarin and dabigatran had higher initial and overall ACT values and needed lower UFH dose to achieve recommended ACT values in comparison to patients on rivaroxaban and apixaban. Further prospective studies to determine initial UFH doses as well as target ACT values, depending on oral anticoagulants used, are required.

## CONFLICT OF INTEREST

The authors have no conflicts of interest to declare.

## Data Availability

Data available on request from the authors
